# Development of F1 hybrid population and the high-density linkage map for European aspen (*Populus tremula* L.) using RADseq technology

**DOI:** 10.1186/s12870-017-1127-y

**Published:** 2017-11-14

**Authors:** Anatoly V. Zhigunov, Pavel S. Ulianich, Marina V. Lebedeva, Peter L. Chang, Sergey V. Nuzhdin, Elena K. Potokina

**Affiliations:** 1 Saint Petersburg State Forest Technical University, Institutskiy per, 5, 194021 St. Petersburg, Russia; 2 Vavilov Institute of Plant Genetic Resources (VIR), Bolshaya Morskaya, 42-44, 190000 St. Petersburg, Russia; 3 University of Southern California, Los Angeles, CA 90089 USA; 4 Saint Petersburg State University, Universitetskaya emb. 7/9, St. Petersburg, 199034 Russia

**Keywords:** European aspen, Intra-specific cross, Full-sibling population, RADseq, High resolution linkage map, SNP, Growth-related traits, QTL mapping

## Abstract

**Background:**

Restriction-site associated DNA sequencing (RADseq) technology was recently employed to identify a large number of single nucleotide polymorphisms (SNP) for linkage mapping of a North American and Eastern Asian *Populus* species. However, there is also the need for high-density genetic linkage maps for the European aspen (*P. tremula*) as a tool for further mapping of quantitative trait loci (QTLs) and marker-assisted selection of the *Populus* species native to Europe.

**Results:**

We established a hybrid F1 population from the cross of two aspen parental genotypes diverged in their phenological and morphological traits. We performed RADseq of 122 F1 progenies and two parents yielding 15,732 high-quality SNPs that were successfully identified using the reference genome of *P. trichocarpa*. 2055 SNPs were employed for the construction of maternal and paternal linkage maps. The maternal linkage map was assembled with 1000 SNPs, containing 19 linkage groups and spanning 3054.9 cM of the genome, with an average distance of 3.05 cM between adjacent markers. The paternal map consisted of 1055 SNPs and the same number of linkage groups with a total length of 3090.56 cM and average interval distance of 2.93 cM. The linkage maps were employed for QTL mapping of one-year-old seedlings height variation. The most significant QTL (LOD = 5.73) was localized to LG5 (96.94 cM) of the male linkage map, explaining 18% of the phenotypic variation.

**Conclusions:**

The set of 15,732 SNPs polymorphic in aspen and high-density genetic linkage maps constructed for the *P. tremula* intra-specific cross will provide a valuable source for QTL mapping and identification of candidate genes facilitating marker-assisted selection in European aspen.

**Electronic supplementary material:**

The online version of this article (10.1186/s12870-017-1127-y) contains supplementary material, which is available to authorized users.

## Background

*Populus* species are the fast-growing trees appreciated worldwide due to their rapid growth and quality of wood, which is amenable to a variety of mechanical and chemical processing. They are of particular importance for the pulp and paper industries and the fuel and energy sectors, providing valuable raw materials in a short time. In Europe, the most frost-resistant and productive *Populus* species that has adapted to unfertile and acidic soils is aspen (*Populus tremula* L.) [1]. Despite its value and advantages, aspen has a major drawback - massive trunk damage by heart rot caused by the fungi *Phellinus tremulae* (Bond.) Bond. et. Boriss. Aspen clones with the highest growth rate (i.e. natural triploids) have been reportedly less affected by the heart rot pathogen [1, 2]. Therefore, the priority tasks of the aspen breeding programs in Russia have been growth rate, resistance to diseases and winter hardiness, which in turn, are closely related to the phenology traits [3].

The timing of bud flush and bud set are important adaptive traits which represent critical trade-offs between survival and growth [4]. Too early bud flush or late bud set increases the risk of frost damage, whereas too early bud set might be disadvantageous in the competition for resources with other trees during the short growing season characteristic of northern latitudes [5]. The knowledge about the genetic architecture of the adaptive and grow-related traits will allow effective breeding of the most valuable commercial aspen clones.

Breeding in woody perennial species is a long-term process where the availability and application of genomic tools would have obvious benefits. Several remarkable genomic resources for *Populus* breeding were recently made available, e.g. the assembled genome sequence of a *P. trichocarpa* [6], a 34 K SNP array available for genotyping of natural populations of *Populus trichocarpa* and other *Populus* species [7], and a number of genetic linkage maps of different species in the genus (reviewed in [8]). The linkage maps allowed identification of QTLs related to valuable traits followed by candidate gene identification and developing gene-specific markers that should facilitate breeding in long-lived perennial plants like *Populus*. For example, the linkage maps developed for full-sib F1 progenies of interspecific crosses between *P. tremula* x *P. tremuloides* allowed identification of QTLs related to sex determination in aspen [9]. Consequently, the sex-linked genomic region was reviewed considering gene loci located in congruent genome region of the sequenced *P. trichocarpa*. These genes were bioinformatically evaluated for their potential as candidate genes for sex determination in aspen, suggesting as a target Potri.019G047300 (‘TOZ19’). A PCR-based marker was subsequently developed for determination of the sex of non-flowering aspen individuals or seedlings [10].

Technologies such as next-generation sequencing (NGS) provide new inspiring opportunities to obtain genome-wide genetic markers for high resolution linkage map construction. Recently, Restriction-site Associated DNA Sequencing (RADseq) was employed to identify a large number of SNPs for linkage mapping of F1 progenies derived from an interspecific cross of North American *P. deltoides* and *P. simonii*, a native tree species widely distributed in northern China. 2545 high quality SNP markers allowed construction of two parent-specific linkage maps with an average distance of ~3 cM between adjacent markers, facilitating extensive QTL mapping studies in poplar [11]. Using the same F1 hybrid population of *P. deltoides* × *P. simonii* Mousavi et al. [12] demonstrated a strategy for constructing dense genetic linkage maps in hybrid forest trees by combining RADseq and whole-genome sequencing technologies.

So far only inter-specific *Populus* crosses were assessed for the high-resolution linkage mapping, although some *Populus* species with huge distribution area would provide the large diversity of phenotypic traits that is comparable with variability observed among the different species [3]. In Europe two native *Populus* species have a sufficiently wide geographical distribution to be considered for studies of natural variation: black poplar (*P. nigra*) and aspen (*P. tremula*) [5].

The distribution area of *P. tremula* covers Western and Eastern Europe, the European part of Russia, the Crimea, the Caucasus, Siberia, the Far East, Kazakhstan, Central Asia, Mongolia, China, the northern part of the Korean peninsula and Algeria [1]. Inhabiting a wide range of environmental conditions, aspen exhibits variability in numerous morphological and physiological traits. To analyze the natural phenotypic and genetic variation, the Swedish Aspen (SwAsp) Collection was established to reveal genetic determinants underlying genotypic variation of traits important for adaptation [5]. Investigation of the aspen germplasm collection elucidated exceptionally high levels of phenotypic and genotypic variation in aspen and exposed high levels (>0.55) of broad-sense heritability of phenology traits such as bud set, bud burst, and onset of senescence. Remarkably, a significant correlation between bud set and growth was reported [13]. The availability of a comprehensive set of SNP markers with known position on the aspen linkage map would facilitate the future identification of genes controlling adaptive traits.

The objective of this study was to construct high-density genetic linkage maps for the European aspen (*P. tremula*) using a comprehensive set of SNP markers generated with RADseq data. Herein, double digest RADseq (ddRADseq) method [14] was used for the genotyping purposes. The ddRADseq technique was earlier reported as an efficient and low-cost method for de novo SNP discovery and genotyping of a non-model species compared to the existing RADseq approaches [14, 15]. The linkage maps would provide a tool for identifying quantitative trait loci (QTLs) underlying the variation of forestry-important biological traits such as fast growth, resistance to heart rot, wood quality, root-cropping ability, phenology and winter hardiness.

## Methods

### Mapping population

To construct a high resolution linkage map an F1 full-sib mapping population was developed from the intra-specific cross of two aspen genotypes (*P. tremula*). Both male and female trees grew in the same location in the St. Petersburg area at a distance of only 8 m from each other. The parental trees had comparable ages: 30–32 years for the female and 28–30 years for the male tree, based on the number of the annual rings estimated by taking a core sample. The height of the female tree was h = 19.8 m and the diameter at breast height was d = 0.27 m. For the male tree those measurements were estimated as h = 17.6 m and d = 0.23 m, correspondingly. Inhabiting the same environment, both parents had similar dendrometry records, except for phenological traits and winter hardiness. Ten and 6 days delay were recorded for the female tree compared to the male tree for generative bud flush and vegetative bud flush, respectively. As a consequence, serious frost damage of twigs was registered for the male parent, whereas no frost damage was recorded for the female tree.

Controlled crossing was performed in April 2016 in a greenhouse of St. Petersburg State Forest Technical University. Freshly cut twigs of male and female aspen about 1 m long were placed in a 10-l clean plastic jug with water. After 10 days of incubation at room temperature (20 °C), catkins emergence was observed for both male and female twigs. Hybridization was conducted by artificial pollination of female flowers using a small brush with pollen collected from male catkins.

Next, twigs with pollinated female catkins were bagged to exclude random airborne pollen. After 14 days female catkins produced small fruit splitting to release lots of tiny, cottony seeds. The seeds were immediately planted in small plastic containers (12x10x7cm) filled with wet peat substrate. Germination occurred within a couple of days after the planting (Additional file 1: Figure S1). After 2 weeks the seedlings were transferred into individual containers and placed in lining-out nursery (Additional file 1: Figure S2). In May 2016, the seedlings reached the height of approx. 50 cm and were re-planted into larger individual containers (9x9x11 cm). By September 2016 about 1100 containerized seedlings were ready for the field.

The seedling stock was then divided into three parts to establish the F1 full-sibling aspen plantations in three diverse environments. 192 seedlings were planted in September 2016 in a field 70 km southwest of Saint Petersburg (59°16′33″N, 30°08′32″E). This location is classified as a humid continental climate; distinct moderating influence of the Baltic Sea cyclones result in warm, humid and short summers and long, moderately cold wet winters. Another set of 192 seedlings was transported to West Siberia and planted in a field nursery in Surgut (61°15′00″N, 73°25′00″E). This environment features a continental subarctic climate with long and very cold winters (daily mean temperature in January is (20 °C) and short but relatively warm summers. The third part of the containerized seedlings was used to establish the hybrid aspen plantation in the South of Russia (Voronezh, 51°40′19″N, 39°11′03″E). The Saint Petersburg population of F1 hybrids was used to construct the high-density linkage map.

### DNA isolation

DNA was extracted from dried leaves collected from parental trees and each of 122 one-year progeny seedlings. For each sample 0.15 g of dried leaf material were frozen in liquid nitrogen and grinded with Mixer Mill MM 400 (Retsch, Germany). Grinded samples were washed with fresh washing buffer: 100 mM Tris-HCl, pH 8.0; 50 mM EDTA; 1 M NaCl; 1% 2-mercaptoethanol; 1% polyvinylpyrrolidone (PVP) (k-30, S_ABC_) [16]. DNA extraction was carried out using a DNA Extran-3 kit (Syntol, Moscow) according to the manufacture protocol. Extracted DNA was cleaned with Genomic DNA Clean & Concentrator kit (Zymo Research).

### Construction and sequencing of ddRAD libraries

Six ten double-digested RADseq (ddRADseq) libraries were constructed for the two parental genotypes (8 library replicates for each parent) and their 122 progenies (1 library per individual) according to the protocol described by Plekhanova et al. [17]. Concentration of DNA for each individual sample was normalized to 10 ng/ul and digested for 1 h at 37°C with 0.7 units of *HindIII* (NEB, USA) in a 20-ul reaction volume. Digestion was stopped by incubating the reaction mixture at 65°C for 20 min. Next, the “barcode” adapter was ligated to the end generated by *HindIII,* allowing pooling of the samples. The ligation reaction was carried out at 22°C for 1 h in a volume of 50 ul with 1.6 units of T4 ligase (NEB, USA). T4 ligase inactivation was reached by heating at 65°C for 10 min. Next, 10 ul for each sample were pooled and simultaneously purified with an Agencourt AMPure XP kit. The second restriction digestion was performed with 0.7 units of *NlaIII* (NEB, USA) in a 20-ul reaction volume at 37°C for 1 h with subsequent inactivation at 65°C for 20 min. The second “common” adapter was ligated to the overhanging end of *NlaIII*. The reaction mixture volume (50 ul) was dispersed into 14 aliquots and PCR was performed on the 14 aliquots to amplify the DNA fragments. PCR mix (50 ul) contained 1× HF buffer, 25 uM of each primer, 12.5 uM dNTPs and 1 unit of Phusion® High-Fidelity DNA Polymerase (NEB, USA). PCR conditions were: 98°C for 30 s, 14 cycles of 98°C for 10 s, 65°C for 30 s, 72°C for 15 s, then 72°C for 2 min. All 14 PCR reactions were pooled and purified with Agencourt AMPure XP kit. Concentration and size distribution of the prepared libraries were checked by automated electrophoresis with 2100 Bioanalyzer (Agilent Technologies, USA). Fragments were sequenced as 150 base reads on an Illumina HiSeq2500 at the University of Southern California Genome and Cytometry Core. Illumina data is available at NCBI under the BioProject PRJNA395596.

### SNP calling

Illumina reads were mapped to the *P. trichocarpa* reference genome [6] using BWA MEM [18] under default mapping parameters. Polymorphisms were called using the GATK pipeline [19] which considers indel realignment and base quality score recalibration, and calls variants across all samples simultaneously through the HaplotypeCaller program in GATK. Variants were filtered using standard hard filtering parameters according to GATK Best Practices recommendation [20, 21]. More precisely, GBS data was filtered to only retain SNP calls with Mapping Quality (MQ) > 37 and Quality by Depth (QD) > 24. Both metrics take into consideration the quality of the mapping and genotype calls to ensure that only those with highest confidence were used. The SNPs were also filtered to retain those with MQRankSum < |2.0|, which ensure that there was no difference in the Mapping Quality scores for both alleles. This filtering retained those that pass all three criteria. Lastly, VCFtools software [22] was used to implement the following inclusion criteria: minor allele frequency (MAF) more than 3%, genotype call-rate more than 90%, and Hardy-Weinberg Equilibrium (HWE) exact *P*-value more than 10^−5^.

### Linkage map construction

A RADseq linkage map of aspen was constructed using the pseudo-testcross mapping strategy [23], where a mapping population is developed by hybridizing two unrelated highly heterozygous parents to produce a set of F1 progeny [15]. With this strategy the maternal and paternal linkage maps were constructed separately using linkage phase inferred between adjacent markers.

A linkage map was constructed using JoinMap 3.0 [24]. The linkage group assignments were made under the logarithm of odds (LOD) score limit of 5.0. The regression mapping algorithm and Kosambi’s mapping function were used for map construction with the following settings: Rec = 0.4, LOD = 1.0, Jump = 5. The resulting linkage maps were drawn by JoinMap 3.0 output.

### QTL mapping

Composite Interval Mapping (CIM) analysis was implemented using Windows QTL Cartographer v.2.5 [25] with a 2-cM walk speed and a type-I error rate of 5%. Intervals of five background markers with a window width of 10 cM were analyzed to control the QTL background effects. The Kosambi mapping function was chosen for recalculation of maps on genotype data. The LOD significance threshold was determined by a permutation test with 1000 permutations at a significance level of *P* < 0.05. The QTL results were drawn using a custom Perl script, which is available upon request.

## Results

### Construction and sequencing of ddRAD libraries

A total of 138 ddRAD libraries were constructed from the two parents (each in 8 replicates) and their 122 F1 offsprings. The 138 ddRAD libraries were sequenced to generate 236.56 million reads, comprising approximately 33.83 Gb of sequencing data (Additional file 2: Figure S3). The female and male parental data sets contained 20.17 million reads (comprising 2.88 Gb) and 13.08 million reads (1.87 Gb), respectively. From the 122 offspring, 204.108 million reads containing 29.07 Gb of data were produced for SNP detection. 82.62% and 81.89% of reads were successfully mapped to the *P. trichocarpa* reference genome for the female and male parent, respectively. For the 122 progenies the average percentage of mapped reads was 77.51%.

### SNP genotyping

GATK identified 16,234 SNPs to further analysis after passing through filtering criteria (see Methods). Using these SNPs, 122 progenies were screened for their individual ancestry by maximum likelihood estimation using the ADMIXTURE software [26]. The number of ancestral populations was K = 2, corresponding to 8 reps of each female and male parent (Additional file 3: Figure S4). 7 out of 122 F1 offsprings showed unclear ancestry and were excluded from the further analysis, whereas 115 progenies perfectly demonstrated their hybrid nature and were used for the linkage map construction.

Next, chi-square tests were performed for each of 16,234 SNPs to check for correspondence to Mendelian segregation ratios 1:1, 1:2:1 and 1:1:1:1 within the 122 F1 progenies. Those SNPs deviating seriously from the Mendelian ratios (*p* > 0.05) were excluded from the analysis. There were SNPs showing no polymorphism between the 2 *P. tremula* parents, but polymorphic when compared to the *P. trichocarpa* reference. Those SNPs were considered as potentially valuable markers and were included in the final list of 15,732 robust SNPs useful for genotyping purposes in aspen (Additional file 4).

### High-resolution genetic map construction

To construct the female map SNPs from the 15,732 robust SNPs list were selected if they i) were maternal heterozygous (ab) and paternal homozygous (aa), ii) showed segregation ratio 1:1 among the progeny, iii) showed clear polymorphism when 8 reps of maternal genotype and 8 reps of paternal genotype were compared, iiii) had less than 10% of missing data across the entire F1 population. Based on these criteria, 1000 SNPs were chosen for female map construction. Correspondingly, 1055 SNPs that were paternal heterozygous (ab) and maternal homozygous (aa) passing through the above mentioned criteria were selected to develop the male linkage map (Table 1).

**Table 1:** Number of SNPs used for developing of the female and male *P. tremula* linkage maps

Female genotype^a^	Male genotype	SNPs in parents	SNPs used for mapping
ab	aa	2429	1000
aa	ab	2426	1055
ab	ab	2229	0
aa	bb	8584	0
ab	ac	64	0
Total		15,732	2055

^a^a, b, c denote up to four possible alleles from two parents at an SNP site

Due to the high level of synteny and collinearity between the linkage maps of different *Populus* species and physical map of *P. trichocarpa*, [11, 12, 27] we divided the SNPs into 19 preliminary groups according to their distribution across the 19 chromosomes of the reference genome of *P. trichocarpa*. SNP markers were then assembled separately for each of 19 chromosomes for the male and female map using default parameters of JoinMap 3.0. For the best mapping order of the SNP markers on a chromosome and precise estimation of the genetic distances, F1 offsprings showing more than 10% of missing data for a particular linkage group were excluded from the mapping procedure. Therefore, the number of F1 individuals involved in SNP mapping for each chromosome varied between 99 to 115.

**Fig. 1:**
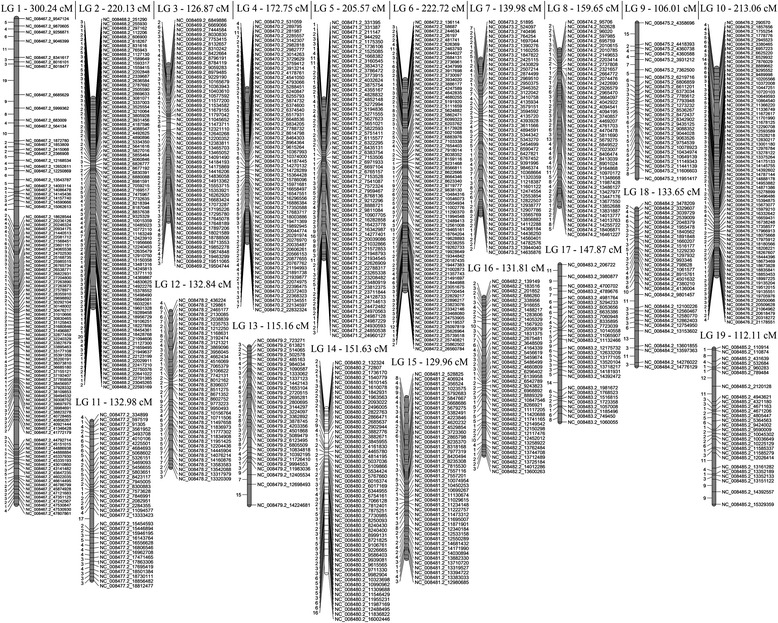
Maternal linkage map constructed for *P. tremula* intra-specific cross using the JoinMap 3.0. software. Genetic distances (cM) between neighboring SNPs are given on the left side of the linkage groups. SNP marker names are denoted by the names and positions of chromosomes of the reference genome *P. trichocarpa*

**Fig. 2:**
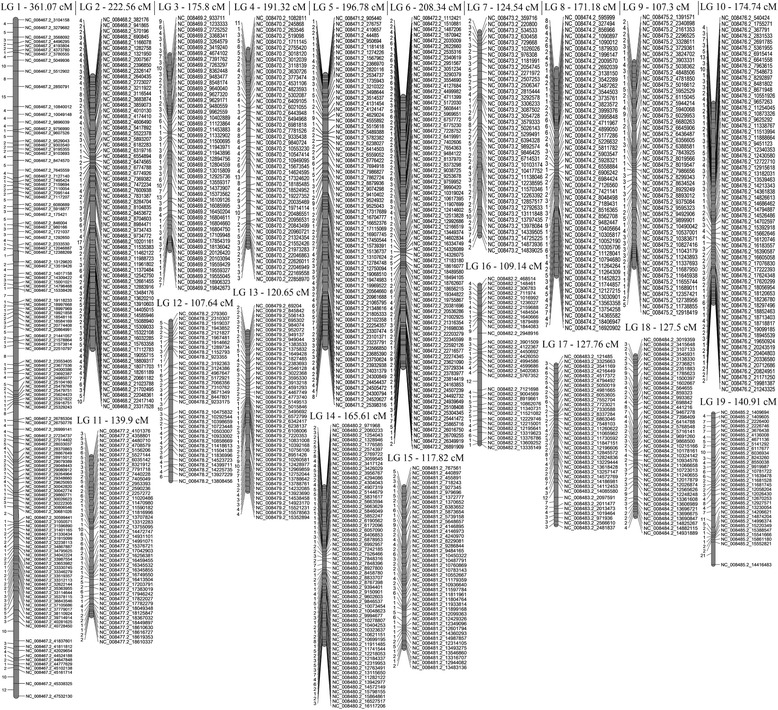
Paternal linkage map constructed for *P. tremula* intra-specific cross using the JoinMap 3.0. software. Genetic distances (cM) between neighboring SNPs are given on the left side of the linkage groups. SNP marker names are denoted by the names and positions of chromosomes of the reference genome *P. trichocarpa*

For the resulting maternal map 1000 SNPs were assigned to 19 linkage groups at the LOD threshold of 10.0 comprising a total genetic distance of 3054.99 cM. The length of single LG ranged from 106.01 cM (LG9) to 300.24 cM (LG1), with an average interlocus distance of 3.05 cM (Table 2). The corresponding paternal map represented a total length of 3090.56 cM and ranged from 107.3 cM (LG9) to 361.07 cM (LG1), with an average distance between neighboring markers of 2.93. There was a good consistency between the two parental maps in terms of length for the 19 linkage groups with the total correlation coefficient 0.91. The highest similarity was detected for LG2 and LG9, the deepest discordance was revealed for LG3. The two parental linkage maps are presented by Figs. 1 and 2; the detailed information on genetic distance and linkage phase between adjacent SNP markers is listed in Additional files 5 and 6.

**Table 2:** Number of mapped SNPs and genetic distances of *P. tremula* female and male linkage groups (LG)

*P. tremula* (female)			*P. tremula* (male)			Chromosome size (Mb)^a^
Group	SNP number	Length (cM)	Group	SNP number	Length (cM)	
LG1	104	300.24	LG1	133	361.07	50.5
LG2	88	220.13	LG2	73	222.56	25.26
LG3	56	126.87	LG3	48	175.8	21.82
LG4	63	172.75	LG4	46	191.32	24.27
LG5	70	205.57	LG5	74	196.78	25.89
LG6	80	222.72	LG6	87	208.34	27.91
LG7	53	139.98	LG7	39	124.54	15.61
LG8	48	159.65	LG8	52	171.18	19.47
LG9	28	106.01	LG9	57	107.3	12.95
LG10	73	213.06	LG10	65	174.74	22.58
LG11	37	132.98	LG11	46	139.9	18.5
LG12	40	132.84	LG12	34	107.64	15.76
LG13	31	115.16	LG13	47	120.65	16.32
LG14	53	151.63	LG14	64	165.61	18.92
LG15	45	129.96	LG15	45	117.82	15.28
LG16	43	131.81	LG16	33	109.14	14.49
LG17	30	147.87	LG17	37	127.76	16.08
LG18	31	133.65	LG18	43	127.5	16.96
LG19	27	112.11	LG19	32	140.91	15.94
Total	1000	3054.99		1055	3090.56	

^a^Genome size refers to the reference genome of *P. trichocarpa* [6]

**Fig. 3:**
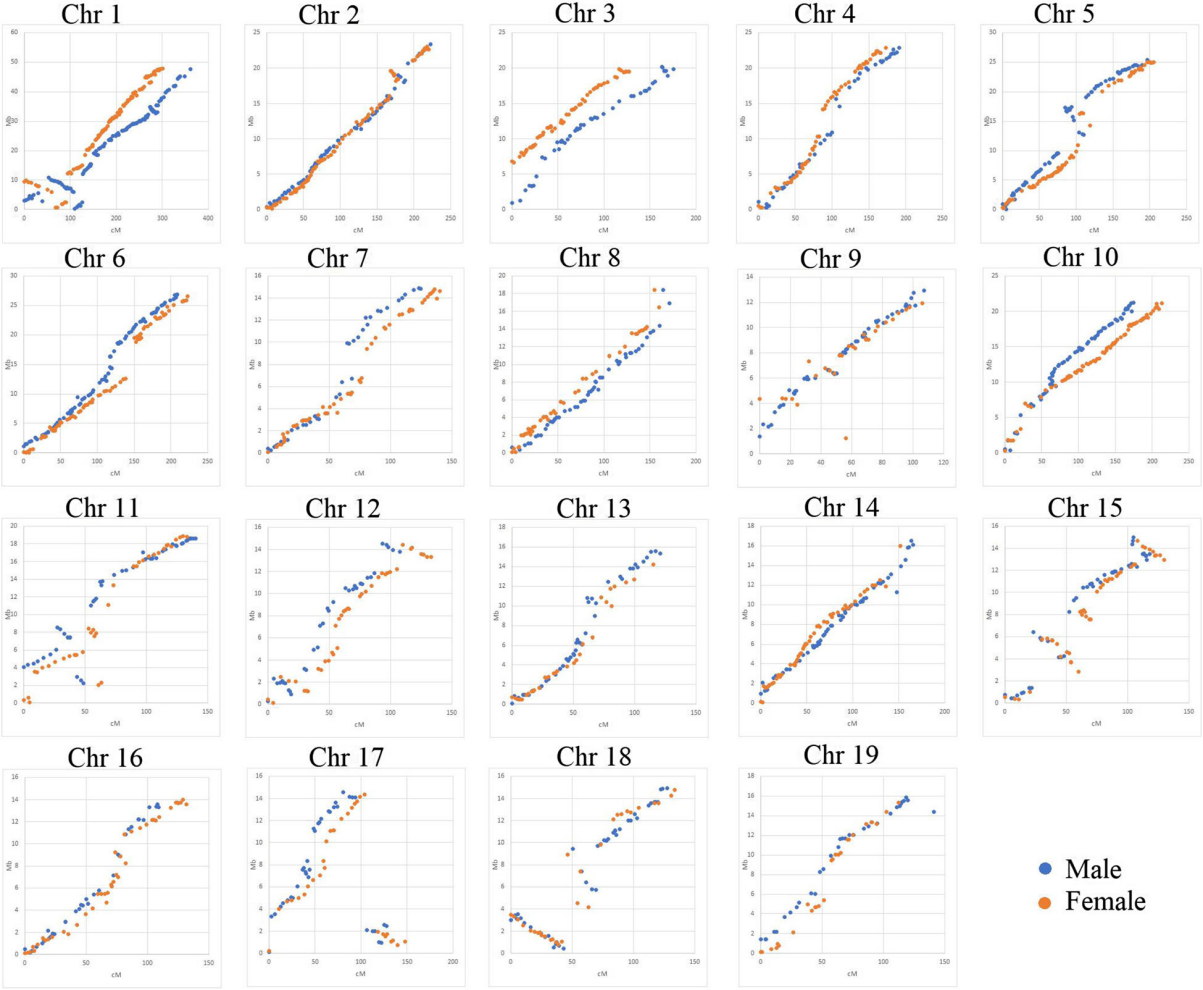
Collinearity between maternal (red) and paternal (blue) linkage maps of *P. tremula* and physical map of *P. trichocarpa.* Genetic position of SNPs on the linkage maps of *P. tremula* are given in cM on the X-axis, while corresponding positions of the SNPs on physical map of *P. trichocarpa* are shown in Mb on the Y-axis

We also estimated syntenic relationships between the maternal and paternal linkage maps of *P. tremula* to the physical map of *P. trichocarpa* (Fig. 3). The order of SNP markers in the aspen parental genetic maps and the *P. trichocarpa* genome was highly consistent, showing high levels of synteny between the genomes of the 2 *Populus* species. However, for some linkage groups e.g. Chr 1,11,12,15,17,18 (Fig. 3) a block of SNPs was detected whose genetic distances were not congruent to the respective SNP positions in the chromosomes of *P. trichocarpa.* Since both maternal and paternal maps showed the same mode of the discordancy relatively to the reference genome, it suggests the presence of chromosome inversions, such as those found on Chr 1, 11, 15, 18.

Recently published high resolution linkage maps of *P. deltoides* and *P. simonii* allowed estimating of correlation between the genetic length of the 19 chromosomes of both the species and physical chromosome size of *P. trichocarpa* [11]. In the present study we developed the high density genetic maps for *P. tremula*. Thus, it was worthwhile to estimate the correlation between the chromosome sizes of all four *Populus* species (Table 3). The highest correlation (0.98) was detected between the genetic map of the *P. tremula* male parent and the physical map of *P. trichocarpa*, suggesting that the linkage map is quite accurate in marker ordering. The correlation coefficients between genetic lengths of linkage groups on the male *P. tremula* map and in the maps of *P. deltoides* and *P. simonii* were nearly identical (0.927 and 0.929 respectively).

**Table 3:** The correlation between genetic length of 19 linkage groups on the maternal and paternal *P. tremula* genetic maps and physical length of *P. trichocarpa* chromosomes

	*P. tremula* maternal map	*P. tremula* paternal map	*P. trichocarpa* ^*a*^	*P. deltoides* ^*b*^	*P. simonii*
*P. tremula* maternal map	1.000000				
*P. tremula* paternal map	0.912140	1.000000			
*P. trichocarpa*	0.911496	0.980897	1.000000		
*P. deltoides*	0.853154	0.926683	0.914898	1.000000	
*P. simonii*	0.899007	0.928977	0.921623	0.939383	1.000000

^a^Chromosome size refers to the reference genome of *P. trichocarpa* [6]

^b^Genetic length of linkage groups of *P. deltoides* and *P. simonii* refers to the corresponding high resolution genetic maps [11]

### Employing of the constructed high resolution genetic map for QTL mapping of growth-related traits in aspen

**Fig. 4:**
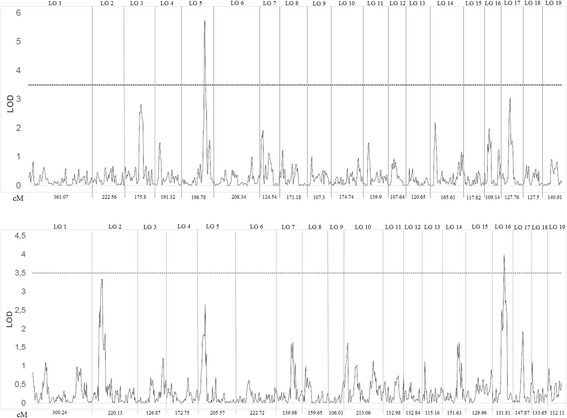
Mapping of QTLs affecting the height variation of one-year-old seedlings derived from artificial cross of two *P. tremula* genotypes. Upper scan: paternal linkage map. Lower scan: maternal linkage map. Length of 19 linkage groups (cM) are given below. LOD significance threshold were determined on the basis of 1000 permutations at a significance level of *P* < 0.05

The F1 mapping population deriving from the intra-specific cross of two *P. tremula* genotypes was developed to monitor phenotypic trait variation in aspen under different environmental conditions. To aid in that that, experimental planting of F1 progenies were established in three different geographical locations facilitating further QTL mapping studies. Since in 2017 only one-year-old seedlings of the F1 population were available for phenotype assessment, we estimated the plant height variation of the seedlings before they were planted in the field (Additional file 7: Figure S5). Among the hybrids of the mapping population, the height of seedlings varied extensively from 15 cm to 127 cm, following a normal distribution (*p* = 0.5450) (Additional file 7: Figure S6). The estimated significant threshold for the seedling height QTLs was determined using a permutation test as LOD = 3.4 (*P* < 0.05). The most significant QTL (LOD = 5.73) was localized to LG5 (96.94 cM) of the male linkage map, explaining 18% of the phenotypic variation (Fig. 4). The nearest co-segregated markers were NC_008471.2_15136737; NC_008471.2_13107624 and NC_008471.2_12784748. Another significant QTL related to one-year-seedling height variation was identified on LG16 of the female map at 70.84 cM in an interval between SNPs NC_008482.2_6139958 and NC_008482.2_6542789. Its contribution to the phenotypic variation was estimated as 15%.

## Discussion

In the present study we first report the high-resolution linkage map for European aspen (*P. tremula*). This is the most widespread *Populus* species in the Northern hemisphere and harbors substantial genetic diversity both within populations and across the species range. Levels of nucleotide polymorphism for many nuclear genes of *P. tremula* were reported to be 2- to 10-fold higher than those in other woody, long-lived perennial plants, such as *Pinus* and *Cryptomeria*, and 2 to 5-fold higher than those in *Betula pendula.* Remarkably, the estimated population subdivision is low, thus, a substantial fraction of the aspen species-wide diversity can be sampled from single populations [28].

Due to the rapid decay of LD in *P. tremula,* it was previosuly assumed that the number of markers needed to ensure adequate genome coverage for QTL mapping purposes may be too high to be feasible in this species. On other hand, the rapid decay of LD suggests that it may be possible to map functional variation to very fine scales in *P. tremula* [28]. The limitation in the number of markers was overcome with the development of NGS technologies (e.g. RADseq [29]), so currently one could take the advantage of the low LD in aspen to achieve fine mapping and candidate genes identification for *P. tremula*.

There are numerous potential targets for the QTL fine mapping in aspen. The huge natural intraspecific genetic variation of *P. tremula* is reflected in the phenotypic diversity and constantly attracts the attention of forest researchers. At least 10 *P. tremula* varieties have been described in the last century just by the shape of leaves (var. *monticola, parvifolia, grandifolia, rotundifolia, minor, oxyodonta, stricta, laevigata, pendula, supine*) and two varieties have been reported based on bark color difference: the very ordinary light-colored *P. tremula* var. *vulgaris* and the dark-colored *P. tremula* var. *fisca* that occurs occasionally in the middle and southern regions of Russia [30]. Phenological races were also designated as intraspecific taxa: aspen with late bud flush (*P. tremula* L. var. *tardifolia* N. Rubtz.) was described from Trans-Ili Alatau (Tien Shan) [31] and reported for Belarus [32]. The late bud flushing variety showed 10–15 days delay compared to an ordinary early aspen. Revising the aspen intra-specific diversity A.Tzarev [3] reported 3% of occurrence of the late bud flushing variety in the forest-steppe regions of Russia (Voronezh area). There, bud flush delay varied from 6 to 14 days depending on the weather conditions in spring, with the late form requiring effective temperatures 1.7–2-fold higher than the early form of aspen. Remarkably, the inspection of plantations of early and late-bud flushing varieties over 30 years old showed that the percentage of trees affected by heart rot is less in plantations of the late form in approximately 1.5 times than in plantations of early aspen [3]. Special attention was traditionally paid to aspen clones with natural resistance to heart rot. Beginning from the nineteenth century small fragmentary natural populations of healthy aspen trees were regularly described within forests dramatically damaged by *Phellinus* pathogen in the Central part of Russia. Similarly, groups of 50–60 trees in otherwise infected aspen forests of Northwest Russia produced coppice shoots at late age (80–90 years), with no any visible signs of fungi destruction. It was intuitively concluded that resistance to the *Phellinus* pathogen in aspen is an inherited trait suitable for breeding [1].

In this paper we report a set of 15,732 SNP markers that are polymorphic between two genotypes of *P. tremula*. This is only a subset of the nucleotide polymorphism in aspen and does not include those regions in *P.tremula* without homology to the *P. trichocarpa* reference genome. About 20% of reads were not mapped to the reference, thus, part of the polymorphism in aspen is still left unexplored. 30% of the SNPs that mapped to the *P. trichocarpa* reference genome produced heterozygous genotypes in the maternal and paternal aspen trees assessed in this study. This is in good concordance with previous reports showing 29.2% of loci in *P. trichocarpa* accessions carrying heterozygous genotypes [7]. The developed set of 15,732 SNP markers would contribute to further association mapping studies in aspen.

Two thousand fifty five SNP markers were successfully employed for the construction of high-resolution maternal and paternal linkage maps for an F1 full-siblings *P. tremula* population. The constructed map comprised 19 linkage groups based on 1055 SNPs for the male map and 1000 SNPs for the female map. The estimated lengths of 3054.99 and 3090.56 cM for the two parental genetic maps were slightly shorter in comparison with the total genetic lengths reported earlier for the high density map of *P. deltoides* (4057 cM) and *P. simonii* (3.809 cM) [11]. On other hand, these values were larger than those reported in *P. adenopoda* (2443.2 cM) and *P. alba* (2719.5) [27] based on SSR and SRAP markers. The genetic lengths of the aspen linkage groups in our study were, however, consistent with the chromosome size of *P. trichocarpa* (Tables 1, 2).

Despite the highly conserved synteny and collinearity observed between SNP order on the chromosomes of *P. tremula* and *P. trichocarpa*, our results suggest the presence of several chromosome inversions between the two taxonomically remoted *Populus* species. *P. tremula* belongs to the section *Populus* (syn. *Leuce* Duby), whereas balsam poplars *P. trichocarpa* and *P. simonii* are from the same section *Tacamahaca* Spach. Even for *P. simonii* genetic maps, several linkage groups exhibited inverse orders of some SNPs relative to the *P. trichocarpa* genome [11]. Moreover, for nearly all linkage groups of *P. deltoides* and *P. simonii* one or more local regions have been reported where SNP order was inconsistent with reference genome positions [12]. We hypothesize that for some linkage groups of *P. tremula* (e.g. Chr 1, 11, 15, 18) chromosome inversions could be exposed relatively to the SNP marker order on *P. trichocarpa* chromosomes. An inversion occurs when a chromosome breaks at two points and the segment bounded by the breakpoints is reinserted in the reversed orientation [33]. A key evolutionary effect of inversions is that they suppress recombination as heterozygotes. As a result, inversions may contribute to reproductive isolation, local adaptation and speciation in plants [34]. The comparison of the whole genome sequencing of *P. tremula* with the reference genome of *P. trichocarpa* would make it possible to determine whether the observed inconsistency in the order of SNP markers on two genomes is due to the inversion of chromosomes, rather than the distortion of the genetic map.

## Conclusions

In the present study we describe a set of 15,732 SNPs polymorphic within *P. tremula,* a plant that has the widest-known geographic distributions of any tree species. The SNPs were obtained by RADseq genotyping of two parental aspen trees and their 122 F1 progenies followed by mapping of 20.17 million Illumina reads to the *P. trichocarpa* reference genome. 2055 of the SNP markers were successfully employed for the construction of high-resolution maternal and paternal linkage maps of the intraspecific aspen cross. The constructed linkage maps were successfully employed for mapping of QTLs related to plant height variation in the full sibling family. The high resolution linkage map and the set of SNP markers will provide a valuable source for QTL mapping and association studies in *P. tremula* facilitating marker-assisted selection in European aspen.

## Additional files

Additional file 1: Figure S1. Germination of hybrid seeds (F1) obtained via artificial crossing of two parental aspen genotypes. **Figure S2.** Two-weeks-old F1 hybrid aspen seedlings placed in lining-out nursery. (PPTX 1375 kb)

Additional file 2: Figure S3. Information about 138 sequenced ddRAD libraries and reads successfully mapped to the *P. trichocarpa* reference genome. (PDF 391 kb)

Additional file 3: Figure S4. Individual ancestry of 122 F1 aspen offspring estimated by maximum likelihood method based on 16,234 SNPs with ADMIXTURE software. (JPEG 1846 kb)

Additional file 4: List of 15,732 robust SNPs available for aspen genotyping. (TXT 8857 kb)

Additional file 5: Detailed information on genetic distances and linkage phase between adjacent SNP markers in the maternal linkage map constructed for *P. tremula* intra-specific cross. (XLSX 427 kb)

Additional file 6: Detailed information on genetic distances and linkage phase between adjacent SNP markers in the paternal linkage map constructed for *P. tremula* intra-specific cross. (xlsx, 428 kb) (XLSX 463 kb)

Additional file 7: Figure S5. Evaluation of one-year-old seedling height variation before the plants were planted in the field. **Figure S6.** Chi-square goodness of it test for Normality of seedling height values among the 122 F1 progenies derived from *P. tremula* intra-specific cross. (PPTX 939 kb)
