# Supplementary material for: Development of F1 hybrid population and the high-density linkage map for European aspen (Populus tremula L.) using RADseq technology
Source: BMC Plant Biol. 2017 Nov 14;17(Suppl 1):180. doi: 10.1186/s12870-017-1127-y (PMC5688504; doi:10.1186/s12870-017-1127-y)
Supplement: Supplementary file 1 — Germination of hybrid seeds (F1) obtained via artificial crossing of two parental aspen genotypes. Figure S2. Two-weeks-old F1 hybrid aspen seedlings placed in lining-out nursery. (PPTX 1375 kb) [file 12870_2017_1127_MOESM1_ESM.pptx]

## Slide 1
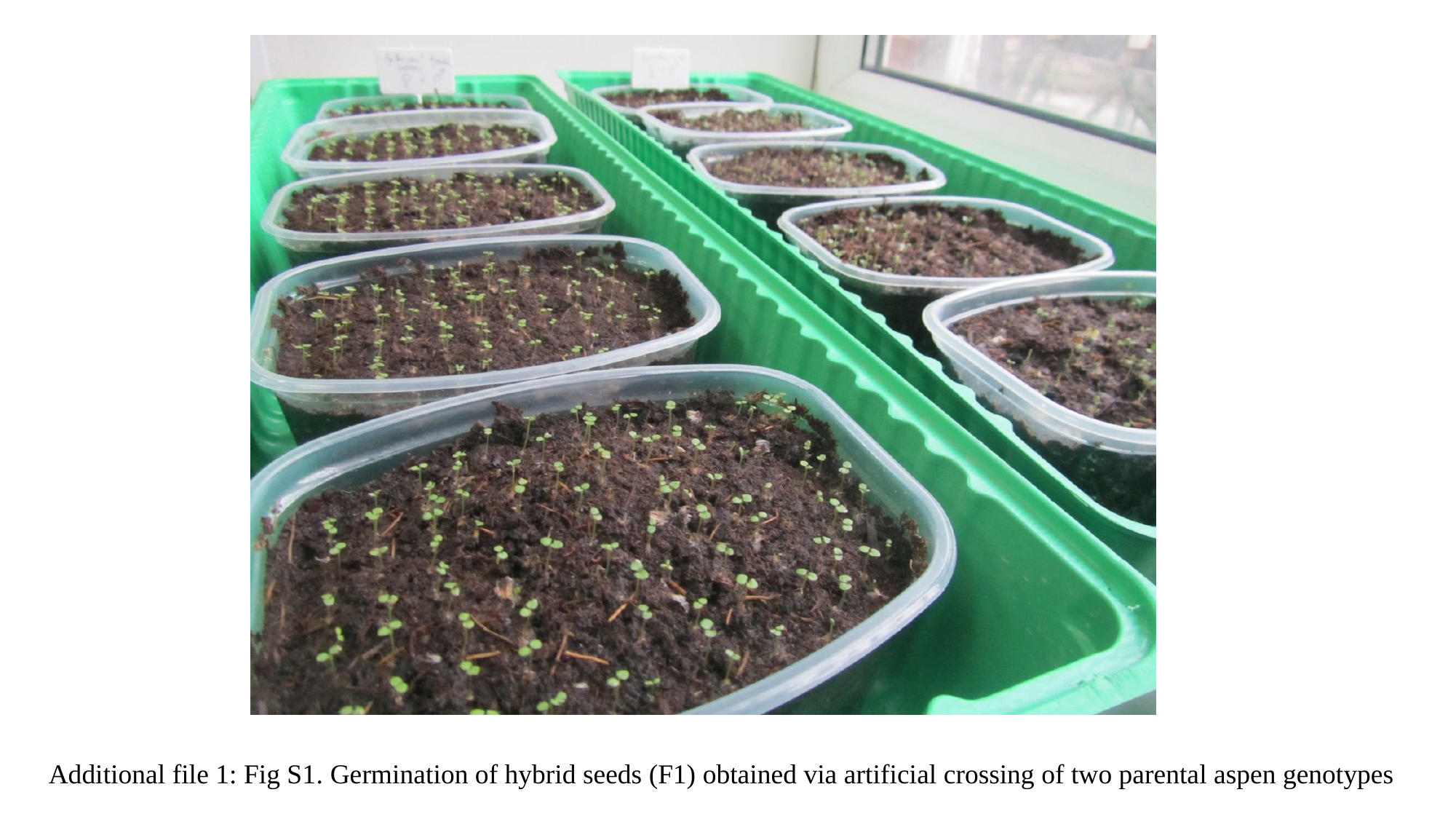

Additional file 1: Fig S1. Germination of hybrid seeds (F1) obtained via artificial crossing of two parental aspen genotypes

## Slide 2
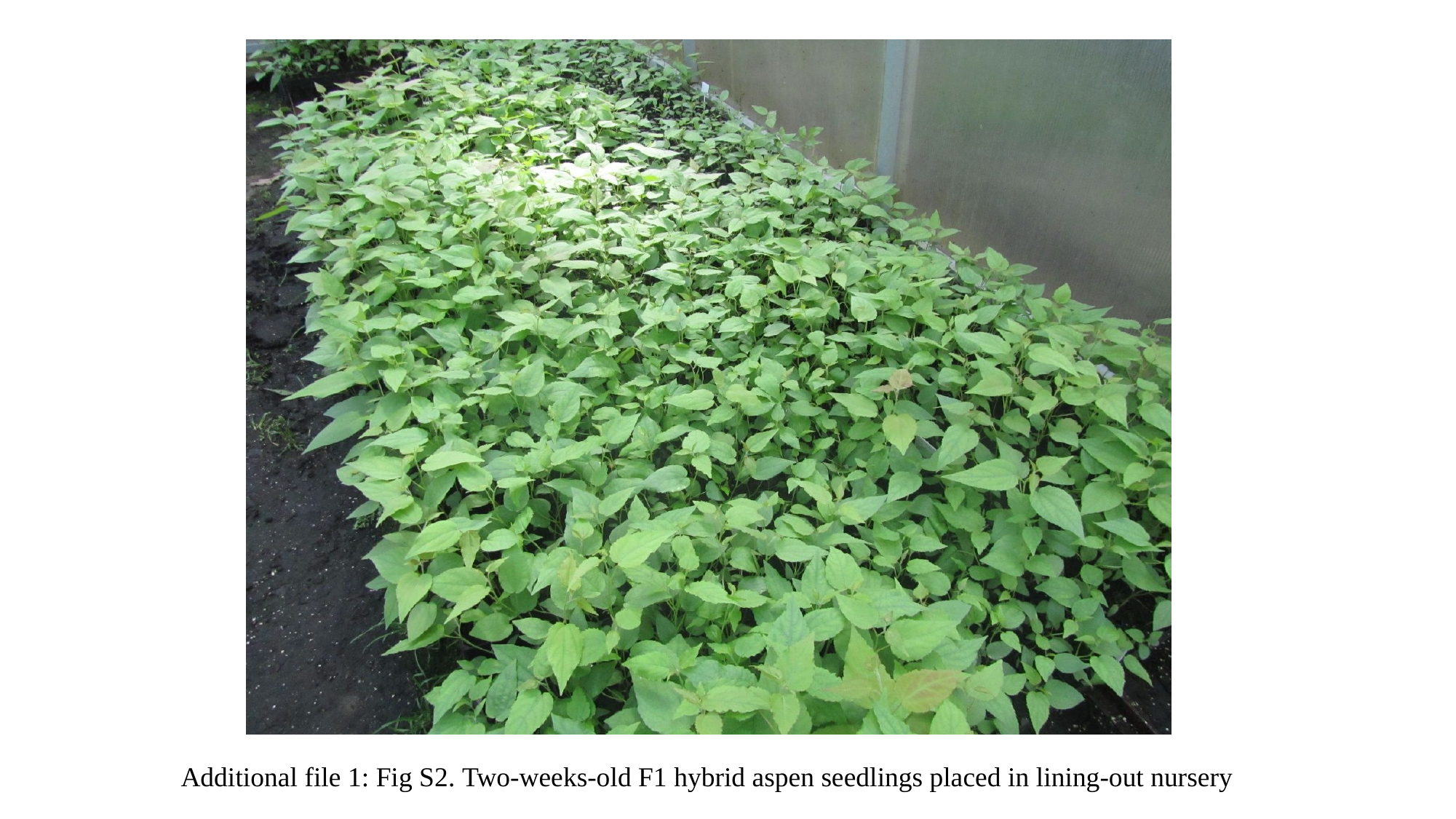

Additional file 1: Fig S2. Two-weeks-old F1 hybrid aspen seedlings placed in lining-out nursery
