# Supplementary material for: Development of F1 hybrid population and the high-density linkage map for European aspen (Populus tremula L.) using RADseq technology
Source: BMC Plant Biol. 2017 Nov 14;17(Suppl 1):180. doi: 10.1186/s12870-017-1127-y (PMC5688504; doi:10.1186/s12870-017-1127-y)
Supplement: Supplementary file 2 — Information about 138 sequenced ddRAD libraries and reads successfully mapped to the P. trichocarpa reference genome. (PDF 391 kb) [file 12870_2017_1127_MOESM2_ESM.pdf]

ID of parents and F1 progeny of *P.tremula* hybrid population

P.t. ♂	P.t. ♂	P.t. ♂	P.t. ♂	P.t. ♂	P.t. ♂	P.t. ♂	P.t. ♂	P.t. ♀	P.t. ♀	P.t. ♀	P.t. ♀
P.t. ♀	P.t. ♀	P.t. ♀	P.t. ♀	II-3-8	II-3-9	II-3-10	II-3-10a	II-3-11	II-3-14	II-3-15	II-3-18
II-3-19	II-3-20	II-3-23	II-3-24	II-3-25	II-3-26	II-3-27	II-3-28	II-3-29	II-3-31	II-3-39	II-3-40
II-3-43	II-3-44	II-3-59	II-3-61	II-3-63	II-3-64	II-3-65	II-3-66	II-3-68	II-3-72	II-3-73	II-3-74
II-3-75	II-3-76	II-3-77	II-3-78	II-3-79	II-5-2	II-5-3	II-5-4	II-5-8	II-5-11	II-5-12	II-5-13
II-5-14	II-5-15	II-5-17	II-5-20	II-5-21	II-5-22	II-5-23	II-5-25	II-5-27	II-5-30	II-5-31	II-5-32
II-5-33	II-5-34	II-5-36	II-5-40	II-5-42	II-5-43	II-5-45	II-5-48	II-5-51	II-5-52	II-5-54	II-5-59
II-5-61	II-5-62	II-5-72	II-5-78	II-5-80	II-1-37	II-1-66	II-1-78	II-1-81	I-3-3	I-3-6	I-3-9
I-3-12	I-3-15	I-3-27	I-3-31	I-3-32	I-3-34	I-3-38	I-3-42	I-3-45	I-3-50	I-3-53	I-3-55
I-3-56	I-3-58	I-3-59	I-3-60	I-3-66	I-3-67	I-3-70	I-3-71	I-3-72	I-3-75	I-3-77	I-3-78
I_4_02_48	I-4-12	I-4-15	I-4-19	I-4-21	I-4-26	I-4-28	I-4-35	I-4-39	I-4-48	I-4-51	I-4-65
I-4-73	I-4-76	I-4-78	I-4-79	I-4-81	II-81						

Number of reads per plant

409819	580746	2495935	2339984	2095526	2652722	1142205	1362847	1309498	1125397	4951747	2781051
3158641	2063907	1563679	3214875	162419	1667320	1223804	3624830	2141321	1559517	849363	2461566
2233784	2436085	2244530	3091497	1855034	2959190	1886385	1851545	2904143	2205750	1663915	2443913
2573774	1291049	1591703	1156330	1530481	1234046	1398216	2134153	1573845	1481356	2158497	2015753
2007059	1213641	1730450	2034705	2002824	1414022	725625	1311266	2297534	2524685	1282857	1755467
1308884	1072700	2350523	1092320	879171	1742244	835723	788996	1333653	1483808	1653548	1098858
1688232	1047198	1610343	1759418	906301	1039065	837175	1700042	2178005	1926551	1950924	1656864
1359471	1349508	909113	1289593	329229	85971	237725	688097	1737748	507996	4540424	2497657
892246	1469773	722709	2291345	1285816	2969175	950355	1991108	1632568	1657975	1849268	3876104
2061771	1086891	3060675	2456125	2876104	501126	1738079	1812403	233175	2319115	3317792	1440650
796350	1765722	2136084	2125130	108172	2507219	617068	1728878	1040484	1411759	1243230	1477233
2555343	761703	2847479	925021	2419653	1770924						

Percentage of reads mapped to the *P.trichocarpa* reference genome

0.7745	0.7811	0.8338	0.8512	0.8256	0.8333	0.8263	0.8254	0.7769	0.789	0.8349	0.8243
0.8373	0.8549	0.8577	0.8352	0.8071	0.813	0.8077	0.7566	0.8205	0.7814	0.7383	0.8083
0.816	0.7931	0.8202	0.8144	0.7519	0.8053	0.8101	0.8259	0.776	0.7862	0.8199	0.8229
0.7577	0.7919	0.8047	0.7813	0.7998	0.772	0.7994	0.8291	0.7786	0.791	0.819	0.7856
0.8274	0.7732	0.8069	0.8163	0.7856	0.7648	0.8038	0.8332	0.8318	0.7882	0.7823	0.8055
0.804	0.8021	0.8239	0.7804	0.816	0.7359	0.7863	0.8053	0.8015	0.8116	0.8141	0.8351
0.812	0.7681	0.8191	0.8232	0.7977	0.8085	0.7525	0.7533	0.8304	0.7866	0.788	0.8145
0.814	0.7795	0.8279	0.7963	0.8133	0.8963	0.1442	0.7248	0.7814	0.7496	0.7532	0.7498
0.7274	0.7306	0.6921	0.7555	0.785	0.7698	0.7789	0.7559	0.7266	0.7391	0.78	0.7688
0.78	0.7342	0.7113	0.7599	0.7444	0.7629	0.5554	0.7801	0.7695	0.7669	0.7586	0.7696
0.74675	0.7342	0.7785	0.7492	0.7851	0.7496	0.5584	0.755	0.7578	0.7566	0.7652	0.743
0.7258	0.7661	0.7889	0.7268	0.8362	0.8362						
