# Supplementary figures and images for: Development of F1 hybrid population and the high-density linkage map for European aspen (Populus tremula L.) using RADseq technology
Source: BMC Plant Biol. 2017 Nov 14;17(Suppl 1):180. doi: 10.1186/s12870-017-1127-y (PMC5688504; doi:10.1186/s12870-017-1127-y)

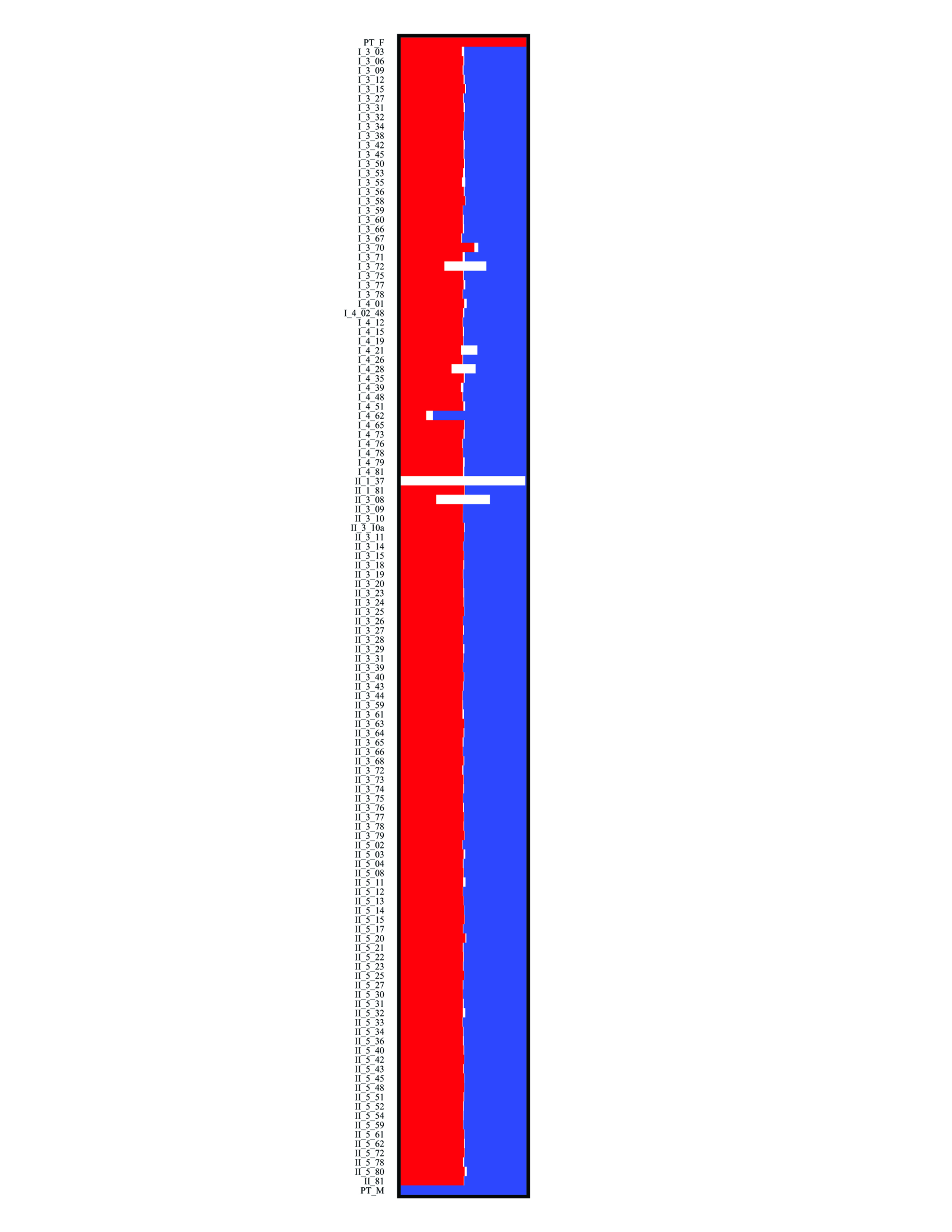

Supplement: Supplementary file 3 — Individual ancestry of 122 F1 aspen offspring estimated by maximum likelihood method based on 16,234 SNPs with ADMIXTURE software. (JPEG 1846 kb) [file 12870_2017_1127_MOESM3_ESM.jpg]
