# Supplementary material for: Development of F1 hybrid population and the high-density linkage map for European aspen (Populus tremula L.) using RADseq technology
Source: BMC Plant Biol. 2017 Nov 14;17(Suppl 1):180. doi: 10.1186/s12870-017-1127-y (PMC5688504; doi:10.1186/s12870-017-1127-y)
Supplement: Supplementary file 7 — Evaluation of one-year-old seedling height variation before the plants were planted in the field. Figure S6. Chi-square goodness of it test for Normality of seedling height values among the 122 F1 progenies derived from P. tremula intra-specific cross. (PPTX 939 kb) [file 12870_2017_1127_MOESM7_ESM.pptx]

## Slide 1
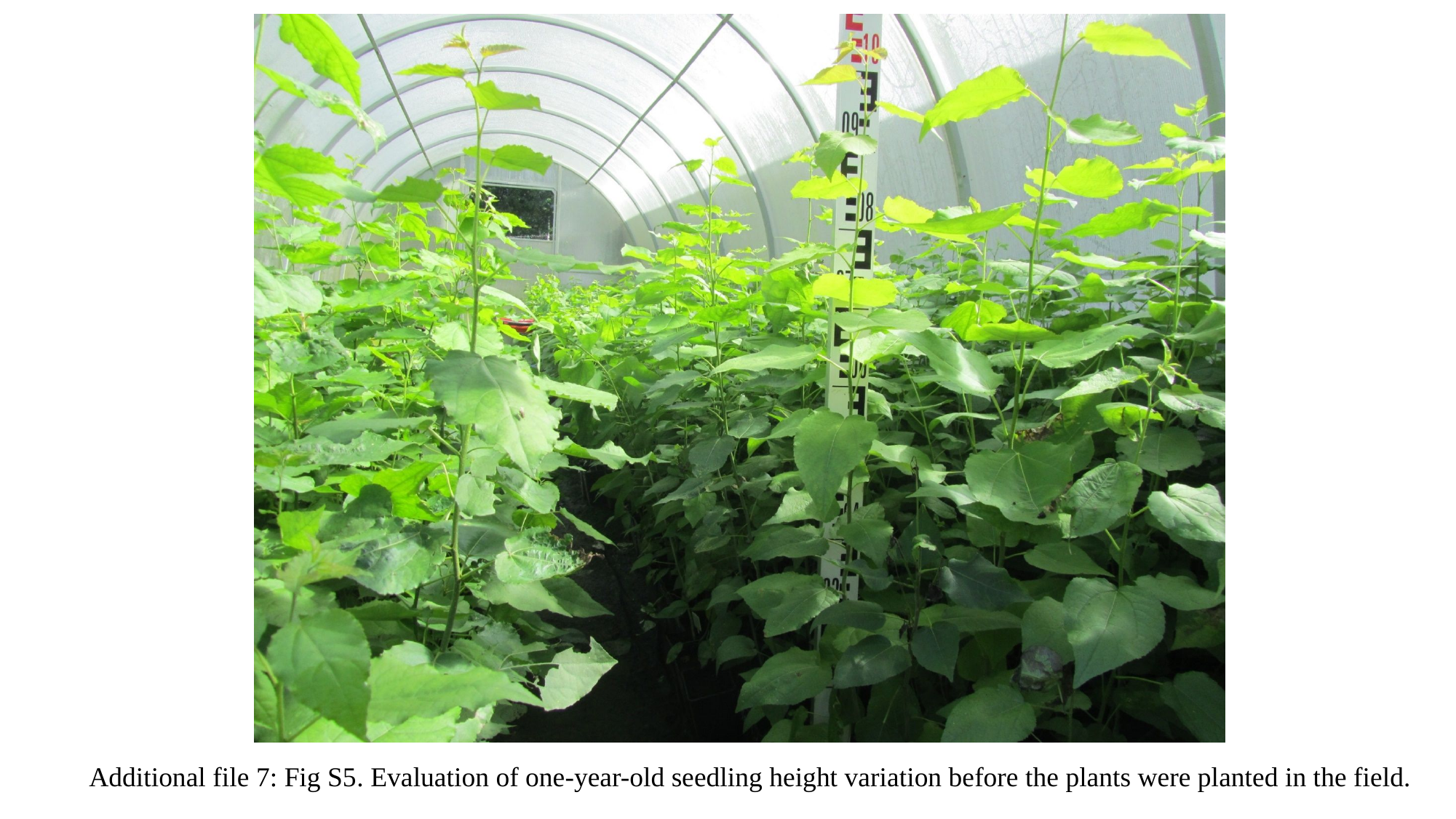

Additional file 7: Fig S5. Evaluation of one-year-old seedling height variation before the plants were planted in the field.

## Slide 2
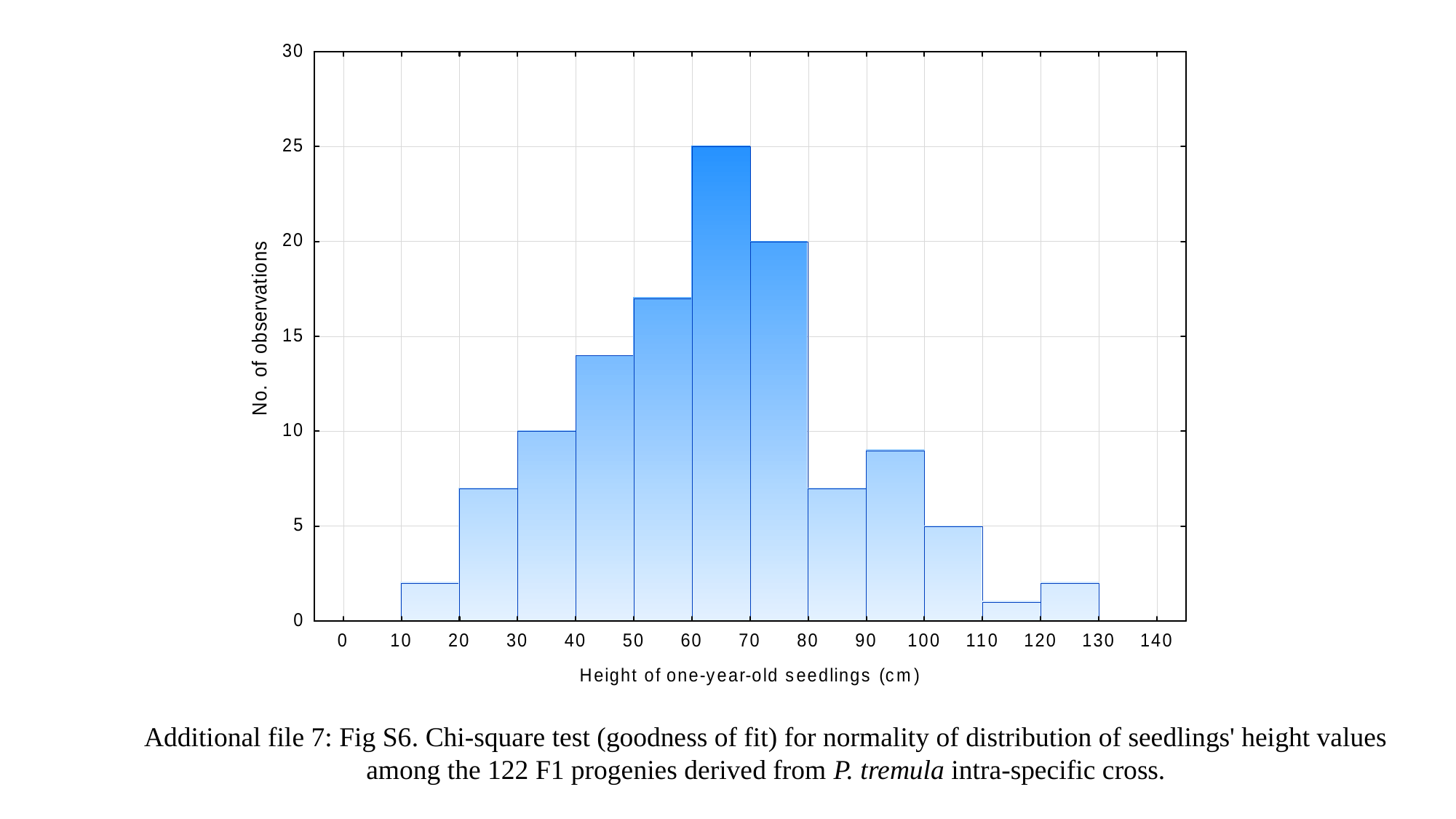

Additional file 7: Fig S6. Chi-square test (goodness of fit) for normality of distribution of seedlings' height values among the 122 F1 progenies derived from P. tremula intra-specific cross.
